# Scale and rate heterogeneity in the EQ-5D-5L valuation

**DOI:** 10.1186/s12955-024-02271-w

**Published:** 2024-07-13

**Authors:** Maksat Jumamyradov, Benjamin M. Craig, Michał Jakubczyk

**Affiliations:** 1 Department of Economics, University of South Florida, Tampa, FL USA; 2 Division of Decision Analysis and Support, SGH Warsaw School of Economics, Warsaw, Poland

**Keywords:** Health valuation, EQ-5D-5L, Scale and discount rate

## Abstract

**Objectives:**

To estimate values on a quality-adjusted life year (QALY) scale using individual preference evidence, choice analyses typically include ancillary parameters, such as scale factors and discount rates. These parameters potentially differ among respondents. In this study, we investigated how allowing heterogeneity in scale and rate affects the estimation of EQ-5D-5L values.

**Methods:**

Using the first wave of the 2016 EQ-5D-5L valuation study (*N* = 1017), we estimated a conditional logit (CL) model and three mixed logit models: random scale, random rate, and bivariate. Prior to the exploratory study, we hypothesized that scale and rate are correlated and that allowing heterogeneity in both parameters decreases the number of insignificant incremental effects. We confirmed the exploratory findings by re-estimating these models using paired comparison responses from a second wave (*N* = 1229).

**Results:**

Scale and rate exhibited significant heterogeneity and were positively correlated. As hypothesized, allowing this heterogeneity improved the face validity of the EQ-5D-5L value set by reducing the number of insignificant incremental effects (from 6 to 2 *p*-values > 0.05; out of 20). Nevertheless, the CL and bivariate mixed logit estimates are highly correlated and concordant (Pearson correlation coefficient of 0.897, Spearman correlation coefficient of 0.888, Lin’s concordance coefficient of 0.763).

**Conclusions:**

Allowing this heterogeneity adds three parameters to the estimation (two variances and a correlation) and improves the face validity of the EQ-5D-5L values. This finding may influence experimental design and choice analysis in health valuation more generally.

## Highlights

1. Allowing heterogeneity in scale and rate improves the estimation of EQ-5D-5L values in terms of face validity, namely, reducing the number of insignificant incremental effects.

2. From the high positive correlation between scale and rate, we can infer that people who heavily discount future outcomes are more sensitive to smaller differences in their net present value on a quality-adjusted life-year (QALY) scale.

3. Controlling for scale and rate heterogeneity slightly reduced the size of the incremental effects, raising the lower bound of the EQ-5D-5L values from -0.545 to -0.191.

## Introduction

In health valuation, the purpose is to estimate preference weights for health outcomes that represent societal values on a quality-adjusted life-year (QALY) scale. On a QALY scale, “immediate death” has a value of 0, and “Starting today 1 year with no health problem then die” has a value of 1. Apart from these two anchors, choice analyses often include ancillary parameters, such as scale factors and discount rates. The primary aim of this paper is to investigate how allowing heterogeneity in scale and rate affects the estimation of EQ-5D-5L values.

In a logistic regression, the scale parameter defines the proportional relationship between the value of the initial QALY and a change in the log-odds of choice. A smaller (larger) scale parameter implies that a larger (smaller) difference in value is necessary to achieve the same change in log-odds of choice. In other words, the scale parameter is an inverse measure of the size of the random component. Varying the scale parameter between individuals implies that some respondents have different sensitivities to the value of the initial QALY [1]. The sources of this scale heterogeneity may be related to their behavior (e.g., attention span) or preferences (e.g., connoisseur) [2]. In health preference research more generally, scaling parameters are estimated in analyses of willingness-to-pay (i.e., monetary scaling) and maximum acceptable risk (MAR).

Apart from the scale parameter, the value of a health outcome depends on temporal discounting. Starting in the 1970s, researchers characterized the value of quality-adjusted life span by simply multiplying quality of life by length of life (i.e., no discounting). However, in the late 2010s, it was shown that discounting may be incorporated into health valuations [3–5]. Discounting is widely accepted in economics and finance; however, some outcome researchers express health-state utilities anchored on “dead” and “full health” and do not account for temporal discounting. In economic evaluations more generally, the marginal utility of time is decreasing (i.e., each additional day is worth less than the prior day), so incorporating discounting into health valuation enhanced its coherence with microeconomic theory [6, 7]. More recently, Karim and colleagues showed how the discount rate may vary within and between latent classes [8]. The sources of rate heterogeneity may be related to the respondents’ perceptions of death (e.g., nontraders) or their marginal decrease in utility of life years.

Prior to the exploratory analysis, we hypothesized that by allowing individual-level randomness in these two ancillary parameters, the estimates of the EQ-5D-5L value set might improve in terms of face validity. The EQ-5D-5L descriptive system has five ordinal domains, each representing increasing severity of health problems. Therefore, we assessed face validity by counting the number of insignificant incremental effects under alternative logit specifications estimated using a first survey wave. To complement this aim, we explored the variances and correlations of these parameters and their implications beyond health valuation.

As recommended by Craig, de Bekker-Grob, González Sepúlveda, and Greene, we confirmed the initial findings using a second wave [9]. The exploratory results led us to further hypothesize that scale and rate are positively correlated at the individual level. For example, the net present value (NPV) of the 10 QALYs depends on the discount rate, but the effect of NPV on the log-odds ratio depends on the scale parameter. Persons who discount heavily (lightly) may seem to be more (less) sensitive to differences in NPV, leading to a positive correlation. Analogously, a person who dislikes spicy foods may seem more sensitive to spice. Although this may now seem intuitive, to the best of our knowledge, no study has produced empirical evidence of this correlation.

The remainder of this paper is organized as follows. Section 2 describes the methods we used in this project, including the theoretical foundation, model specifications, exploratory (i.e., wave 1) and confirmatory (i.e., wave 2) data, and estimation techniques. In Sects. 3, 4 and 5, we provide the results, discussion and conclusions, respectively.

## Methods

### Random utility theory for paired comparisons

The theoretical framework of this choice analysis is based on random utility maximization (RUM) theory. According to RUM theory, the utility function ${U}_{itj}={V}_{itj}+{\varepsilon }_{itj}$ of individual $i=1,\dots N$ for alternative $j=1,\dots ,J$ in choice situation $t=1,\dots T$ can be decomposed into a deterministic part of utility ${V}_{itj}$ (representative utility) and a random part of utility ${\varepsilon }_{itj}$. In paired comparison modeling [10], individual $i$ will choose an alternative $j$ if and only if the probability that the utility associated with alternative $j$ is higher than the utility of its alternative.

1 $$\begin{array}{c}{P}_{itj}=P\left({U}_{itj}>{U}_{itk} \right), \forall \,k\ne j\\ {P}_{itj}=P\left({V}_{itj}+{\varepsilon }_{itj}>{V}_{itk}+{\varepsilon }_{itk}\right), \forall \,k\ne j\\ {P}_{itj}=P\left({\varepsilon }_{itk}-{\varepsilon }_{itj}<{V}_{itj}-{V}_{itk} \right), \forall \,k\ne j\end{array}$$

Choice probabilities are calculated based on a relative measure where the utility of one of the alternatives in the choice set is taken as a reference. To derive the choice probabilities, we need to make distributional assumptions about the random part of utility. The conditional logit (CL) model is derived under the assumption that ${\varepsilon }_{itj}$ is independently and identically distributed (IID) with an extreme value type I (EV1) distribution [11–13]. As a result, the difference between two IID EV1 random error terms ${(\varepsilon }_{itk}-{\varepsilon }_{itj})$ has a logistic distribution with scale parameter $\lambda$. This implies that the choice probabilities of the CL model can be expressed in terms of a logistic distribution with a cumulative distribution function

2 $${{{P}}}_{{{i}}{{t}}{{j}}}=\frac{1}{1+\sum_{{{k}}=1}^{{{J}}}\text{exp}[{{\lambda}}({{{V}}}_{{{i}}{{t}}{{k}}}-{{{V}}}_{{{i}}{{t}}{{j}}})]},\boldsymbol{ }\forall \,\boldsymbol{ }{{k}}\ne {{j}}$$

where $\lambda$ is the scale parameter [14].

### Scale and rate heterogeneity in health valuation

For this study, we extended the CL model (Eq. 2) for health valuation on a quality-adjusted life-year (QALY). By construction, the scale parameter is always positive, $\lambda =\text{exp}(\mu )$, and represents the relationship between log-odds and the value of a health outcome ${V}_{itj}$ on a QALY scale. We specify the value of a health outcome ${V}_{itj}$ as a product of two values representing heath ${V}_{itj}^{H}$ and life years ${V}_{itj}^{Y}$:

3 $${{{V}}}_{{{i}}{{t}}{{j}}}={{{V}}}_{{{i}}{{t}}{{j}}}^{{{H}}}\times {{{V}}}_{{{i}}{{t}}{{j}}}^{{{Y}}}$$

In this paper, we assume that the value of health ${V}_{itj}^{H}=1-{\beta {'}x}_{itj}$, where ${x}_{itj}$ is a vector of 20 incremental indicators of health problems in mobility, self-care, usual activities, pain/discomfort and anxiety/depression (i.e., MO, SC, UA, PD, AD), and $\beta$ is a vector of preference weights on a QALY scale. Its homogeneity is a simplifying assumption for the estimation of a single EQ-5D-5L5L value set that may be relaxed in future work.

More specifically, the value of the health profiles is parameterized using 20 incremental effects (i.e., 5 attributes with 4 levels each), where each effect is caused by a dummy variable representing an incremental change in the level of severity of an EQ-5D-5L attribute. Therefore, we can write

4 $${V}_{itj}^{H}=1-\left(\begin{array}{c}\begin{array}{c}{\beta }_{1}M{O}_{12}+{\beta }_{2}M{O}_{23}+{\beta }_{3}M{O}_{34}+{\beta }_{4}M{O}_{45}+\\ {\beta }_{5}S{C}_{12}+{\beta }_{6}S{C}_{23}+{\beta }_{7}S{C}_{34}+{\beta }_{8}S{C}_{45}+\\ {\beta }_{9}U{A}_{12}+{\beta }_{10}U{A}_{23}+{\beta }_{11}U{A}_{34}+{\beta }_{12}U{A}_{45}+\end{array}\\ {\beta }_{13}P{D}_{12}+{\beta }_{14}P{D}_{23}+{\beta }_{15}P{D}_{34}+{\beta }_{16}P{D}_{45}+\\ {\beta }_{17}A{D}_{12}+{\beta }_{18}A{D}_{23}+{\beta }_{19}A{D}_{34}+{\beta }_{20}A{D}_{45}\end{array}\right)$$

As a criterion of face validity, all 20 incremental effects in vector $\beta$ should be positive since they represent losses in value due to increases in the level of severity of a health condition from the full health profile [14].

For the value of life years ${V}_{itj}^{Y}$, the identity function is commonly assumed to be ${V}_{itj}^{Y}={Y}_{itj}$, where ${Y}_{itj}$ represents life years (i.e., no discounting). However, this functional form does not accurately represent the time preferences of the general population [4, 5]. Individuals usually discount over time; i.e., future outcomes affect choices less than present outcomes. To allow for temporal discounting, we adapt the power function (see 4)

$${V}_{itj}^{Y}={Y}_{itj}^{{\alpha }_{i}}$$

where ${\alpha }_{i}$ is the individual-specific power. On a QALY scale, the value of time ${V}_{itj}^{Y}$ equals 1 when ${Y}_{itj}$ equals 1, regardless of the power ${\alpha }_{i}$, and the identity function (i.e., no discounting) implies that the power is unity, ${\alpha }_{i}=1$.

Apart from restricting the individual-specific scale parameter to be positive, ${\lambda }_{i}=\text{exp}({\mu }_{i})$, we restricted the power ${\alpha }_{i}$ to the unit interval, $0\le {\alpha }_{i}\le 1$. More specifically, we transform the power into a discount rate using the complementary log–log (CLL) function, ${\alpha }_{i}=\text{exp}(-\text{exp}\left({r}_{i}\right))$ which is naturally bounded to the unit interval. At first glance, ${r}_{i}$ has an inverse relationship with ${\alpha }_{i}$, and a lower ${\alpha }_{i}$ implies greater discounting of life years; therefore, a higher rate ${r}_{i}$ implies greater discounting. Future analyses may allow for negative discounting or alternative functional forms [15–17].

### The bivariate distribution of the scale and rate among respondents

Due to limited panel evidence per respondent, it is not feasible to estimate individual-specific scales and rates as fixed effects (i.e., ${\mu }_{i}$ and ${r}_{i}$). Instead, we estimated a conditional logit (CL) model and three mixed logit models. First, we estimated the CL model under homogeneity $({\mu }_{i}=\mu ; {r}_{i}=r)$. Under this specification, all respondents have the same scale parameter and discount rate. In the second and third specifications, we estimated the mixed logit models with random scale and random rate, respectively. We refer to these two mixed logit specifications as “univariate” models because each contains only one normally distributed random parameter.

Finally, in the fourth specification, we estimated a bivariate mixed logit model, including the mean and standard deviation of ${\mu }_{i}$ (i.e., $\mu$ and ${\sigma }_{\mu }$, respectively) and ${r}_{i}$ (i.e., $r$ and ${\sigma }_{r}$, respectively), as well as their correlation. The ancillary parameters vary under a bivariate normal distribution and may be correlated. We assume that ${\mu }_{i}$ and ${r}_{i}$ are normally distributed such that $\left({\mu }_{i},{r}_{i}\right)\sim N({\sigma }_{\mu }^{2},\rho ,{\sigma }_{r}^{2})$ where ${\sigma }_{\mu }^{2}=Var({\mu }_{i})$, ${\sigma }_{r}^{2}=Var({r}_{i})$ and $\rho =Corr({\mu }_{i},{r}_{i})$.

To shed more light on this potential bias, we express the individual-specific ancillary component, ${\lambda }_{i}{V}_{itj}^{Y}$ (apart from the value of health), as an exponential regression with two ancillary parameters (an intercept ${\mu }_{i}$ and a coefficient ${\alpha }_{i})$, where ${Y}_{itj}>0:$

$${\lambda }_{i}{V}_{\mathit{itj}}^{Y}=\text{exp}\left({\mu }_{i}\right){Y}_{itj}^{{\alpha }_{i}}=\text{exp}\left({\mu }_{i}+{\alpha }_{i}ln\left({Y}_{itj}\right)\right)$$

In this study, life years ${Y}_{itj}$ range from 1 to 10 years; therefore, $ln\left({Y}_{itj}\right)$ ranges from zero to 2.303. Given that $ln\left({Y}_{itj}\right)$ is always positive, the ancillary component can increase through either ancillary parameter (${\mu }_{i}$ or ${\alpha }_{i}$). In econometric terms, $ln\left({Y}_{itj}\right)$ is an instrumental variable needed to identify the two ancillary parameters.

### Data

**Fig. 1:**
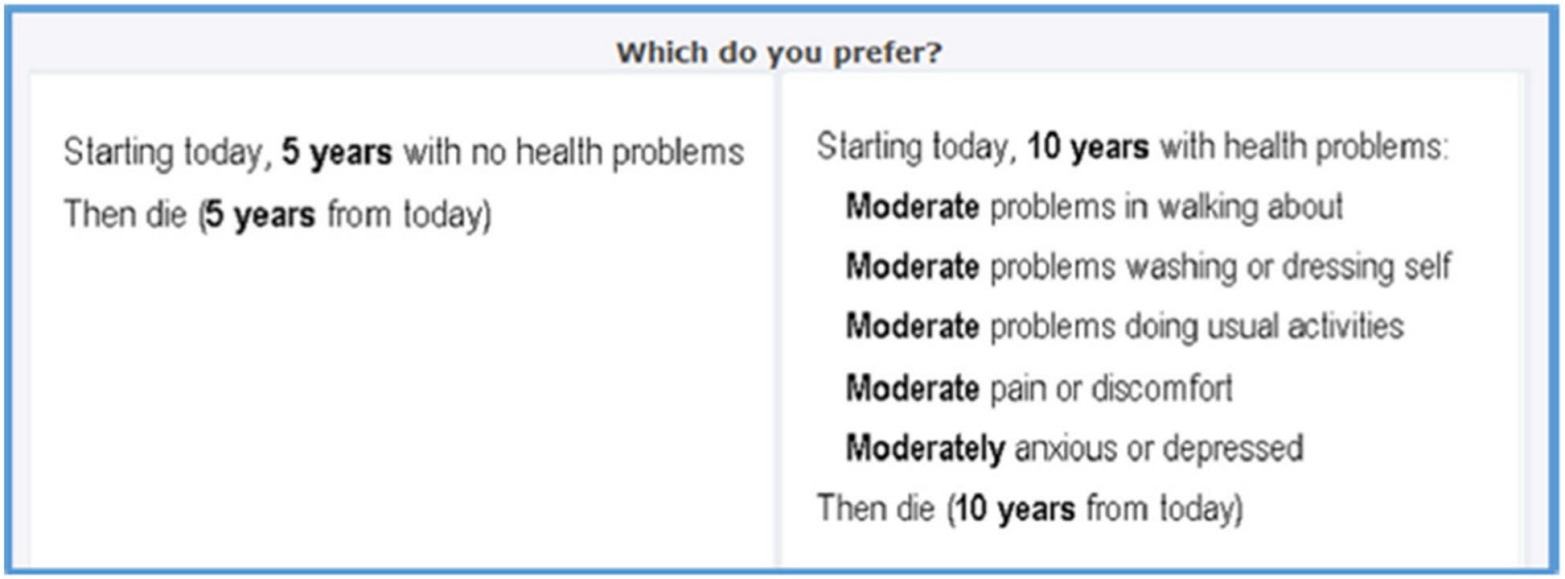
Example of a paired comparison

In 2016, 8,222 U.S. respondents (4074 in wave 1 and 4148 in wave 2) from all 50 states and Washington, D.C., completed an online survey that included 20 paired comparisons. The design of the paired comparisons was largely based on the EuroQol Valuation Technology (EQ-VT v1.0) protocols [18]. An example of the paired comparison conducted in the study is illustrated in Fig. 1. In this paper, we provide a general overview of the study. More details can be found in other studies [3, 19].

Each paired comparison is presented as a variation of health descriptions based on the EQ-5D-5L. The five dimensions (i.e., attributes) of the EQ-5D-5L are mobility, self-care, usual activities, pain/discomfort and anxiety/depression, where each dimension is characterized by five levels ranging from no problems (i.e., level 1) to slight, moderate, severe, and unable/extreme problems (i.e., level 5). For instance, the health description on the right side of Fig. 1 can be represented as a vector of five numbers 33333 since all five dimensions are at a moderate level. For each comparison, respondents were asked, “Which do you prefer?” regarding a pair of alternatives described using the EQ-5D-5L and lifespan attributes.

The online survey consisted of 3160 pairs, 1600 of which are efficient (or “quality only”) pairs and 1560 of which are quantity-quality pairs. In efficient pairs, both health descriptions consisted of varying levels of health problems with the same life years (e.g., 12345 vs 54321). In the quantity-quality pairs, one of the health descriptions involves no health problems (i.e., 11111). Furthermore, 80 out of 1560 quantity-quality pairs included “immediate death”, which represents “dead” pairs, as one of the alternatives. The data were collected in two parts: an exploratory survey consisting of 1560 pairs and a confirmatory survey consisting of 1600 pairs. The survey data were collected at four temporal units (i.e., days, weeks, months, and years). This analysis included only the pairs with year units (1017 respondents in wave 1 and 1229 in wave 2) because the other pairs did not describe events after 1 year (i.e., discounting).

With the diversity of pairs, it is mathematically feasible to identify the scale and rate separately using either wave of this dataset. Imagine a paired comparison with identical lifespans. These pairs may identify differential scales within a population, ${\mu }_{i}$. Imagine a paired comparison with differential life years. These pairs may identify differential scales, ${\mu }_{i}$ and rates, ${r}_{i}$. Apart from its pair types, this dataset is one of the largest national health valuation studies ever conducted [3], has both exploratory and confirmatory waves, and applied quota sampling at the pair level to assure that each pair had a minimum number of respondents along 18 demographic quotas.

### Mixed logit and maximum simulated likelihood

To estimate the mixed logit models, the maximum likelihood (ML) estimator of parameter vector $\theta$ can be utilized when the density of dependent variable ${y}_{i}$ conditional on a vector of independent variables ${x}_{i}$, $f({y}_{i}|{x}_{i},\theta )$, has a closed-form such that

$${\widehat{\theta }}_{N}=\text{arg}\underset{\uptheta }{\text{max}}{\sum }_{i=1}^{N}\text{log}f({y}_{i}|{x}_{i},\theta )$$

where $i=1,\dots ,N$. However, ML is not feasible when $f({y}_{i}|{x}_{i},\theta )$ does not have a tractable closed-form. This can be because the density is specified only conditional on latent variables, which cannot be integrated out. Thus, the MSL estimator is a possible alternative [20, 21]. Suppose $\widetilde{f}({y}_{i},{x}_{i},{u}_{i},\theta )$ is an unbiased simulator of the conditional density $f({y}_{i}|{x}_{i},\theta )$ such that

$$f\left({y}_{i}|{x}_{i},\theta \right)={\text{E}}_{\text{u}}[\widetilde{f}({y}_{i},{x}_{i},{u}_{i},\theta )|{y}_{i},{x}_{i}]$$

where ${u}_{i}$ is an individual-specific latent vector (${\mu }_{i}$ and ${r}_{i}$) whose distribution is known and independent of $({y}_{i},{x}_{i})$. Then, the MSL estimator of $\theta$ is defined as

$${\widehat{\theta }}_{SN}=\text{arg}\underset{\uptheta }{\text{max}}\sum\limits_{i=1}^{N}\text{log}\left[\frac{1}{s}\sum\limits_{s=1}^{S}\widetilde{f}({y}_{i},{x}_{i},{u}_{i}^{s},\theta )\right]$$

where ${u}_{i}^{s}(s=1,\dots ,S)$ are drawn independently for each individual $i$ from the distribution of ${u}_{i}$. The MSL estimator is obtained by replacing the intractable conditional p.d.f. $f({y}_{i}|{x}_{i},\theta )$ with its unbiased approximation based on the simulator $\widetilde{f}({y}_{i},{x}_{i},{u}_{i}^{s},\theta )$. In this study, we estimate the mean and variance of each random parameter as well as their *p*-values [3].

In our MSL estimations of the three specifications of the mixed logit model, we use 250 Halton draws (i.e., $S=250$) [22]. We used the MATLAB programming language for all estimations. More specifically, we began by estimating the CL comparator and three specifications using the wave 1 data, which helped us state our hypotheses more clearly. Afterwards, we re-estimated the models and tested these hypotheses using the wave 2 data. Furthermore, we compare the results between waves and models to assess how allowing heterogeneity in scale and rate affects the estimation of EQ-5D-5L values.

## Results

In this section, we present the results for CL and mixed logit estimation using waves 1 and 2 separately. In Table 1, we compare the CL estimates with the mixed logit estimates where we allow correlations between ${\mu }_{i}$ and ${r}_{i}$. In Table 1A (Appendix), we present the univariate results for the mixed logit estimation with random scale and random rate separately.

**Table 1:** Results for conditional and bivariate mixed logit models

	Conditional Logit		Bivariate Mixed Logit	
*N* = 1017 & 1229	Exploratory	Confirmatory	Exploratory	Confirmatory
$M{O}_{12}$	0.011	0.044^b^	0.014^a^	0.033^b^
$M{O}_{23}$	0.044^b^	0.050^b^	0.044^b^	0.064^b^
$M{O}_{34}$	0.141^b^	0.142^b^	0.110^b^	0.084^b^
$M{O}_{45}$	0.117^b^	0.122^b^	0.069^b^	0.034^b^
$S{C}_{12}$	0.027^a^	0.095^b^	0.025^b^	0.087^b^
$S{C}_{23}$	0.038^b^	0.016	0.025^b^	0.045^b^
$S{C}_{34}$	0.151^b^	0.110^b^	0.108^b^	0.062^b^
$S{C}_{45}$	0.153^b^	0.147^b^	0.098^b^	0.090^b^
$U{A}_{12}$	0.024^a^	0.018	0.015^a^	0.022^b^
$U{A}_{23}$	0.027^b^	0.011	0.024^b^	0.027^b^
$U{A}_{34}$	0.144^b^	0.173^b^	0.119^b^	0.131^b^
$U{A}_{45}$	0.030	0.099^b^	0.027^b^	0.056^b^
$P{D}_{12}$	-0.002	-0.016	0.021^b^	-0.006
$P{D}_{23}$	0.047^b^	0.050^b^	0.034^b^	0.061^b^
$P{D}_{34}$	0.225^b^	0.215^b^	0.161^b^	0.153^b^
$P{D}_{45}$	0.096^b^	0.092^b^	0.048^b^	0.048^b^
$A{D}_{12}$	0.020	-0.022	0.035^b^	0.023^b^
$A{D}_{23}$	0.100^b^	0.046^b^	0.058^b^	0.045^b^
$A{D}_{34}$	0.163^b^	0.168^b^	0.123^b^	0.130^b^
$A{D}_{45}$	0.033^b^	-0.016	0.021^a^	0.000
$\mu (mean)$	0.401^b^	0.062	0.988^b^	0.704^b^
$r (mean)$	-0.196^b^	-0.634^b^	-0.003	-2.051^b^
$\mu (SD)$			0.788^b^	0.998^b^
$r (SD)$			2.858^b^	3.120^b^
$\mu$ and $r$ (corr)			0.836^b^	0.912^b^
$V(55555)$	-0.588^b^	-0.545^b^	-0.180^b^	-0.191^b^
$LL$	-12,225	-14,278	-10,249	-11,890

Notice that the scale parameter is equal to $\lambda =\text{exp}(\mu )$, and the power is equal to $\alpha =\text{exp}(-\text{exp}(r))$ to bound the power between 0 and 1

^a^, ^b^represent significance levels at the 5% and 1%, respectively

### Exploratory results

As shown in Table 1, the exploratory CL results produce three insignificant positive effects (*p*-value < 0.01; $M{O}_{12}$, $U{A}_{45}$ and ${AD}_{12}$) and one insignificant negative effect for $P{D}_{12}$. There are also two additional effects with p-values between 1 and 5% (i.e., $S{C}_{12}$ and $U{A}_{12}$). Furthermore, the CL results suggest that “immediate death” is better than experiencing the worst possible EQ-5D-5L description for 1 year (i.e., $V\left(55555\right)=-0.588$). Since the estimated $\mu$ is 0.401, the scale parameter in the CL model is $\lambda =\text{exp}\left(0.401\right)=1.493$. Similarly, since the estimated $r$ is $-0.196$, the power $\alpha$ in the CL model is $\text{exp}\left(-\text{exp}\left(-0.196\right)\right)=0.439$.

In Table A1 (Appendix), we present the univariate results where we allow for random scale and random rate separately. The standard deviations of ${\mu }_{i}$ and ${r}_{i}$ are 1.232 and 1.325, respectively, suggesting that scale and rate heterogeneity exist. However, the random scale model has insignificant effect (5 with *p*-value > 0.05), i.e., one more than the CL model, while the random rate model has same number of insignificant effects (4 with *p*-value > 0.05). In the exploratory results, allowing for one random parameter increases the log-likelihood, but had little impact on the significance of the effects.

When we allow for heterogeneity in both ancillary parameters (Table 1), there are substantive improvements in the estimated incremental effects. In the bivariate mixed logit results, all 20 effects are positive and significant. The estimated standard deviations for ${\mu }_{i}$ and ${r}_{i}$ are 0.788 and 2.858, respectively, which suggest that both the scale and rate parameters are heterogeneous. Furthermore, we find a strong correlation between ${\mu }_{i}$ and ${r}_{i}$, 0.836 (*p*-value < 0.01).

In the bivariate mixed logit model, “immediate death” is better than experiencing the worst possible EQ-5D-5L description for 1 year (i.e., $V\left(55555\right)=-0.180$); however, this value is closer to zero compared to the CL estimate. Apart from this difference in the lower bound, the twenty incremental effects are highly correlated and concordant between the CL and bivariate mixed logit estimates (Pearson correlation 0.970, Spearman correlation 0.916, Lin’s concordance 0.843). Furthermore, we computed the mean scale and power of the bivariate mixed logit as $\lambda =\text{exp}\left(0.988\right)=2.686$ and $\alpha =\text{exp}\left(-\text{exp}\left(-0.003\right)\right)=0.369$. Therefore, the bivariate mixed logit model produce higher scale and lower power than the CL model.

### Confirmatory results

The confirmatory CL results produce 6 insignificant incremental effects (i.e., $S{C}_{23}$, $U{A}_{12}$, $U{A}_{23}$, $P{D}_{12}$, $A{D}_{12}$, and $A{D}_{45}$), 3 of which are negative (i.e., $P{D}_{12}$, $A{D}_{12}$, and $A{D}_{45}$). Compared to the exploratory CL results, there are the same number of positive insignificant effects and 2 more negative effects. The confirmatory CL results suggest that the value of “immediate death” is better than experiencing the worst possible EQ-5D-5L description for 1 year (i.e., $V\left(55555\right)=-0.545$), which is slightly lower than the exploratory CL estimate ($0.588$). The estimated $\mu$ and $r$ in the confirmatory CL are 0.062 and $-0.634$, respectively. Therefore, the scale and power can be derived as $\lambda =\text{exp}\left(0.062\right)=1.064$ and $\alpha =\text{exp}\left(-\text{exp}\left(-0.634\right)\right)=0.588$. Compared to the confirmatory CL results, the exploratory scale is larger ($1.493$), but its power is smaller ($0.439$).

When we allow for heterogeneity in scale and rate in the confirmatory analysis, there are substantive improvements in the estimated incremental effects. Specifically, in the bivariate mixed logit results, there is only 1 negative (i.e., $P{D}_{12}$) and 1 positive (i.e., $A{D}_{45}$) insignificant incremental effect. We computed the scale and power of the bivariate mixed logit model as $\lambda =\text{exp}\left(0.704\right)=2.022$ and $\alpha =\text{exp}\left(-\text{exp}\left(-2.051\right)\right)=0.879$, respectively, which are higher than those of the CL model. The estimated standard deviations for ${\mu }_{i}$ and ${r}_{i}$ are 0.998 and 3.120, respectively, which suggests that both the scale and rate parameters are heterogeneous. Furthermore, we find a strong correlation between ${\mu }_{i}$ and ${r}_{i}$, 0.912 (*p*-value < 0.01).

In the bivariate mixed logit model, “immediate death” is better than experiencing the worst possible EQ-5D-5L description for 1 year (i.e., $V\left(55555\right)=-0.191$); however, this value is closer to zero compared to the CL estimate ($-0.545$). Apart from this difference in the lower bound, the twenty incremental effects are highly correlated and concordant between the CL and bivariate mixed logit estimates (Pearson correlation 0.897, Spearman correlation 0.888, Lin’s concordance 0.763).

## Discussion

In this paper, we explored and confirmed heterogeneity in scale and rate, their correlation, and their effects on the estimation of EQ-5D-5L values. Allowing heterogeneity in scale and rate improved the EQ-5D-5L value set estimates in terms of face validity, namely, reducing the number of insignificant incremental effects.

A higher discount rate ${r}_{i}$ implies that there is less variability in the net present value of life years. For instance, with a high discount rate, the value of 10 years decreases toward the value of 1 year. A higher scale implies that smaller differences in the value of health have a greater impact on log-odds. In other words, a larger scale parameter means more sensitivity. Since our results suggest that there is a high positive correlation between the scale parameter and the discount rate, we can infer that people who discount the future are more sensitive to smaller differences in the net present value of life years. This important finding may be confirmed in future health valuation studies.

In practical terms, allowing for scale heterogeneity implies that the analyst should also allow for rate heterogeneity (or vice versa) as well as estimate the correlation between scale and rate. However, no econometric package is currently available to facilitate this specification of the mixed logit, which may deter its uptake. In terms of the experimental design and blocking, future studies may assign “dying immediately,” episodes of one-year duration, and multi-year episodes to each respondent. This blocking can aid in the identification of scale and rate heterogeneity. If future studies block accordingly and such a package becomes available, reporting this correlation may become common practice in health valuation.

Although the twenty incremental effects are highly correlated and concordant between the CL and bivariate mixed logit estimates, controlling for scale and rate heterogeneity, reduced the size of the incremental effects, raising the lower bound of the EQ-5D-5L values from -0.545 to -0.191. Although some effects decreased in size, the confirmatory bivariate estimation produced only two insignificant effects ($P{D}_{12}$ and $A{D}_{45}$), which merits further discussion. The incremental effect $P{D}_{12}$ represents the effect of the change from no to slight pain or discomfort. This effect is negative and insignificant in both the conditional logit models as well as the confirmatory bivariate mixed logit, which seems to suggest that U.S. adults are unwilling to sacrifice life years to relieve slight pain or discomfort. Further research is needed to verify this effect. The incremental effect $A{D}_{45}$ represents the effect of the change from severely to extremely anxious or depressed. In two prior papers, Craig and colleagues [23, 24] showed that many U.S. adults prefer “extremely” over “severely” in this domain. This preference inversion contradicts the descriptive system and may be due to the diagnostic implications of severe mental health problems and/or the belief that moods may fluctuate between extrema under normal circumstances. The higher lower bound and two insignificant effects may accurately represent the EQ-5D-5L preferences of U.S. adults.

**Fig. 2:**
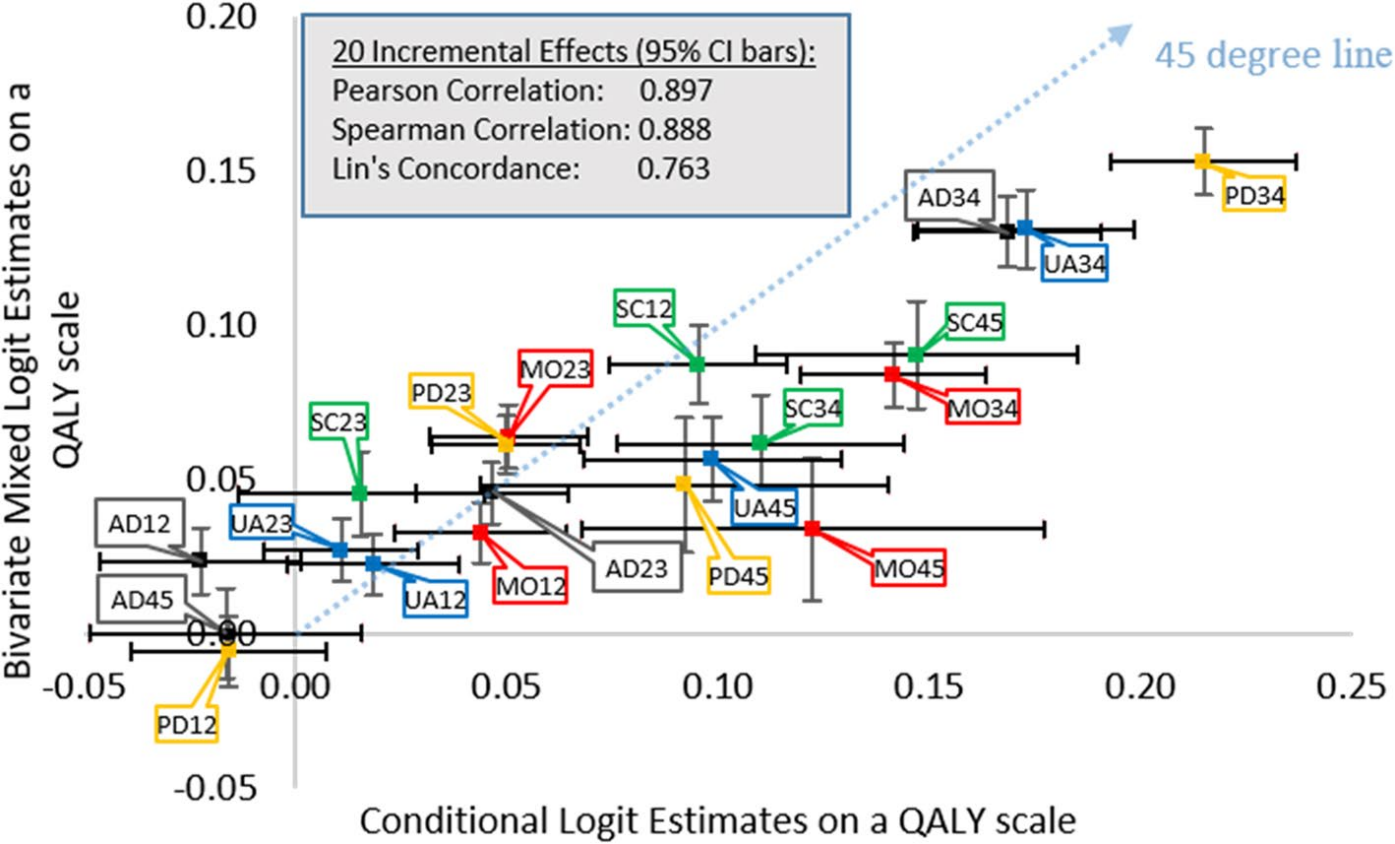
Plot of 20 incremental effects for the conditional logit and bivariate mixed logit

Although the incremental effects of the bivariate mixed logit model appear to be better in terms of sign and significance, they are highly correlated with the CL estimates (Pearson correlation 0.897, Spearman correlation 0.888, Lin’s concordance 0.763). Figure 2 shows the 20 incremental effects from the confirmatory CL and bivariate mixed logit, where incremental effects are color-coded by dimension (i.e., MO: red, SC: green; UA: blue, PD: yellow, AD: black). The differences between the estimates seem to be larger among the more severe effects (from level 3 to 4 or from level 4 to 5).

Alternatively, some analysts may choose to use a hyperbolic discount function instead of a power function to allow for temporal discounting. Craig and colleagues [4] showed that decreasing the marginal value of life span under the assumption of power discounting provides better model fit than alternative functional forms. While Craig and colleagues [4] assumed a homogeneous discount rate (i.e., ${r}_{i}=r$), Jonker and colleagues [5] estimated the mixed logit model with a random hyperbolic discount rate and found strong evidence for non-linear time preferences. In this study, we extended both approaches and estimated a bivariate mixed logit model allowing a correlation between scale and power.

Future analyses may allow for heterogeneity in the incremental effects as well as scale and discount rate parameters, building from these findings. Before the estimation of such a complex model is attempted, we recommend that the authors conduct simulation analyses to verify that they can mitigate the simulation biases. For instance, Jumamyradov and colleagues conducted a simulation study and showed that the mixed logit model can produce biased results even when the model is correctly specified [25]. Nevertheless, we believe that our more parsimonious specification produced reliable results since we found a high correlation both in exploratory and confirmatory datasets.

There are three limitations in our analysis that we would like to mention. First, our mixed logit analysis is based on MSL estimation. This may be problematic for the bivariate specification because Jumamyradov and colleagues [25] showed that the MSL estimator of the mixed logit has difficulty estimating correlations and may produce biased estimates even when correctly specified. Second, we assume only normally distributed random parameters and may consider other distributional assumptions in future research. Third, because of computational capacity constraints, we used only 250 Halton draws in our estimations, which is common place in the literature [22]. Some [26] have shown that increasing the number of Halton draws decreases the simulation bias for bivariate normal and bivariate Poisson-lognormal models.

Our study utilized EQ-5D-5L values from a United States-specific valuation study. While the data source was specific to the U.S., the underlying principles and results of this study are not confined to the U.S. context alone. The methodological approach and findings presented are designed to be broadly applicable and are likely to be generalizable to other settings. Similar methodologies can be applied to different populations and healthcare systems, reinforcing the validity of our approach across diverse settings.

We also would like to point that we acknowledge that the scale heterogeneity is a form of correlation among coefficients in mixed logit models [27, 28]. However, this is not relevant in our study since we are focusing on the correlation between the scale parameter and the discount rate.

## Conclusion

Allowing heterogeneity in rate and scale added three parameters to the conditional logit model (two variances and a correlation) and greatly improved the face validity of the EQ-5D-5L values. We confirmed that persons who highly discount the future are more sensitive to differences in the net present value of QALYs. This intuitive pattern may be confirmed in future EQ-5D-5L valuation studies as well as influence experimental design and choice analysis in health preference research more generally.

## Data Availability

The datasets used and/or analysed during the current study are available from the corresponding author on reasonable request.

## Appendix

**Table:** **Table 1A**. Results for univariate mixed logit models

	Random scale		Random rate	
*N* = 1017 & 1229	Exploratory	Confirmatory	Exploratory	Confirmatory
*MO*_12_	-0.012	0.019^b^	0.003	0.025^b^
*MO*_23_	0.049^b^	0.076^b^	0.045^b^	0.064^b^
*MO*_34_	0.087^b^	0.093^b^	0.103^b^	0.082^b^
*MO*_45_	0.104^b^	0.054^b^	0.049^b^	0.028^a^
*SC*_12_	0.016	0.057^b^	0.022^b^	0.097^b^
*SC*_23_	0.023^a^	0.027^b^	0.020^b^	0.025^b^
*SC*_34_	0.130^b^	0.081^b^	0.101^b^	0.073^b^
*SC*_45_	0.082^b^	0.109^b^	0.089^b^	0.083^b^
*UA*_12_	-0.001	-0.011	0.016^a^	0.013^a^
*UA*_23_	0.018^a^	0.017^a^	0.020^b^	0.027^b^
*UA*_34_	0.127^b^	0.132^b^	0.118^b^	0.126^b^
*UA*_45_	0.030^a^	0.091^b^	0.013	0.067^b^
*PD*_12_	-0.007	-0.043^b^	0.013	-0.020^b^
*PD*_23_	0.035^b^	0.046^b^	0.029^b^	0.062^b^
*PD*_34_	0.155^b^	0.175^b^	0.140^b^	0.142^b^
*PD*_45_	0.124^b^	0.065^b^	0.058^b^	0.034^b^
*AD*_12_	0.009	-0.023^b^	0.030^b^	0.012
*AD*_23_	0.083^b^	0.032^b^	0.060^b^	0.043^b^
*AD*_34_	0.094^b^	0.138^b^	0.101^b^	0.122^b^
*AD*_45_	0.018^a^	0.002	0.014	0.013
*μ (mean)*	0.194	0.228^b^	1.275^b^	0.899^b^
*r (mean)*	-0.227^a^	-0.483^b^	0.422^b^	-0.489^b^
*μ (SD)*	1.232^b^	1.136^b^		
*r (SD)*			1.325^b^	1.320^b^
*V(55555)*	-0.162^b^	-0.134^b^	-0.043^b^	-0.118^b^
*LL*	-11964	-12989	-10666	-12279

Notice that the scale parameter is equal to  and the power is equal to  to bound the power between 0 and 1

^a^, ^b^ represent significance levels at 5% and 1%, respectively

## Abbreviations

MSL: Maximum simulated likelihood
ML: Maximum likelihood
QALY: Quality-adjusted life year
CL: Conditional logit
MO: Mobility
SC: Self-care
UA: Usual activities
PD: Pain and discomfort
AD: Anxiety and depression
EQ-VT: EuroQol Valuation Technology
CLL: Complementary log–log
MAR: Maximum acceptable risk
NPV: Net present value
EV1: Extreme value type 1
IID: Independently and identically distributed
RUM: Random utility maximization
